# 2071. *Strongyloides stercoralis* Testing in a Hospital in Bronx, NYC; A 3-year Experience

**DOI:** 10.1093/ofid/ofad500.141

**Published:** 2023-11-27

**Authors:** Coral N Vargas-Pena, Maria Alyssa Policarpio, Bailey Chan, Michael Kladias, Christina Coyle

**Affiliations:** Jacobi Medical Center/Albert Einstein School of Medicine, Bronx, NY; Jacobi Medical Center/Albert Einstein School of Medicine, Bronx, NY; Jacobi Medical Center, Bronx, New York; NYC Health and Hospitals/Jacobi, New York, New York; Albert Einstein College of Medicine, New York, NY

## Abstract

**Background:**

Once a parasitic infection limited to tropical/subtropical regions, as international travel and immigration continues to rise, Strongyloidiasis is now a disease also prevalent in non-endemic regions, including the United States. Its disease spectrum spans from an asymptomatic infection to a hyper-infection syndrome, predominantly in patients with HTLV-1 and/or on steroids, where it can be deadly if not recognized early.

**Methods:**

Retrospective descriptive study describing patients who tested positive for *Strongyloides stercoralis* between 2019-2023 at Jacobi Medical Center, a safety net hospital in Bronx, NYC. Patients were diagnosed with a serum recombinant immunodiagnostic antigen from *S. stercoralis*-specific larvae.

**Results:**

108 patients were included; the mean age was 53.7+/-16 years, 63% were male. The most common place of birth was Latin America, Caribbean, Mexico in descending order. Prolonged steroid use (more than 1 week) reported by 7.4% and 8.3% had been exposed to chemotherapy/cytotoxic drugs. 92.5% of individuals were asymptomatic. Reason for testing was eosinophilia in 56.5% and migrant screening in 36.1%. The remaining (8%) patients were tested for diarrhea, urticaria and abdominal pain. Infectious diseases performed 76% of testing while primary care tested 15%. Mean time from immigration was 19.9 years. Stool testing was performed in 60 patients; larvae were seen 21.7% of these. Eosinophilia (abs >500/uL) was found in 70.4% of cases. Of the 29.6% w/o eosinophilia, 64.6 % of individuals reported an outdoor toilet. Ivermectin was given to 98% of cases.

**Figure:**
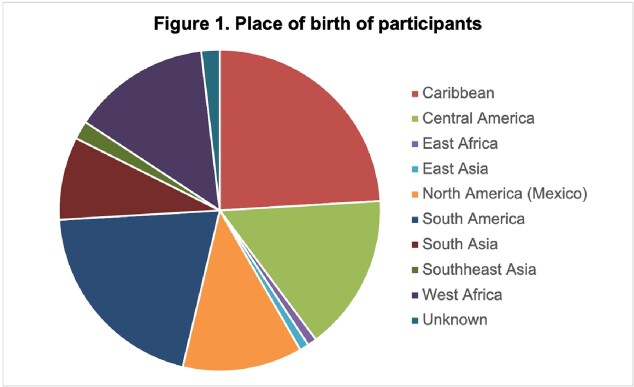

**Figure:**
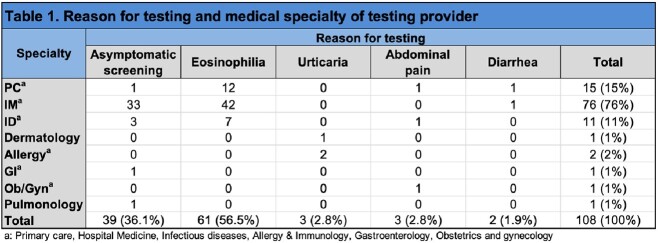

**Conclusion:**

Most cases were asymptomatic and identified on screening. Eosinophilia was seen in 2/3 of cases of strongyloidiasis, but 1/3 did not have eosinophilia. Larvae were reported in 21% of patients who had a stool for O/P underscoring the importance of establishing this diagnosis with serology. In patients without eosinophilia, strongyloidiasis should be considered if living conditions in country of birth put them at risk. As expected, patients had been in the US for many years prior to testing and only 15% of patients were screened by primary care. Increased awareness of the disease must be encouraged, especially amongst primary care physicians.

**Disclosures:**

**All Authors**: No reported disclosures

